# 35.2 PREVENTING PSYCHOSIS: WHAT, (IF ANYTHING) CAN WE LEARN FROM THE EU-GEI INCIDENCE STUDY?

**DOI:** 10.1093/schbul/sby014.148

**Published:** 2018-04-01

**Authors:** Hannah Jongsma, Peter Jones, Craig Morgan, James Kirkbride

**Affiliations:** 1 University of Cambridge; 2 Institute of Psychiatry, Psychology & Neuroscience, King’s College London; 3 University College London

## Abstract

**Background:**

The incidence of psychotic disorders varies across replicable social and environmental gradients at both an individual and a population level, such as higher rates of disorder in urban and migrant populations. However, the factors underpinning this are unclear. The EU-GEI study was established to investigate the incidence as well as the genetic and environmental determinants of first episode psychosis in a multi-national setting. The aim of the present study was to investigate the variance found in the incidence across the 17 catchment areas in the 6 countries (England, France, Italy, the Netherlands, Spain and Brazil) included in this study at both individual and population level, and identify putative predictors of psychosis risk.

**Methods:**

We conducted a population-based study of the incidence of non-organic adult ICD-10 psychotic disorders (F20-F33). Demographic data (age, sex, ethnicity) and OPCRIT diagnoses were collected, and denominator data was estimated from government sources. Crude incidence rates were directly standardised to the 2011 England and Wales Census population to account for population differences in age, sex and ethnicity. Multilevel Poisson regression was carried out to investigate variance in incidence between catchment areas by latitude, population density, and percentage of unemployment, owner-occupied houses and single-person households as markers of catchment-area level social fragmentation, using official government statistics and data from the 2011 European Population and Housing Census.

**Results:**

We identified a total of 2,774 cases over 12.94 million person-years at risk, leading to a crude incidence of 21.4 per 100,000 person-years (95%CI: 19.4–23.4). The age pattern of incidence differed between men and women: crude incidence peaked in men aged 18–24 (61.0 per 100,000 person-years, 95%CI: 59.0–63.1) and declined sharply thereafter, for women rates also peaked in the youngest age group (27.0 per 100,000 person-years, 95%CI: 24.9–29.1) but decline was more gradual and there was a small but robust secondary peak after age 45. By age 35, 68% of male cases had presented to services, compared to 51% of female cases. Age-sex-ethnicity standardised incidence of all psychotic disorders varied 8-fold across settings. Poisson regression revealed higher rates in minority groups (IRR: 1.6, 95%CI: 1.5–1.7), and an association between greater catchment area-level owner-occupancy and lower incidence (IRR for a 10% increase: 0.8, 95%CI: 0.7–0.8). No relationship was found for other putative environmental risk factors, including latitude and population density. Results were similar for non-affective and affective disorders.

**Discussion:**

Variance in treated incidence was substantial and was only partially explained by standardisation for age, sex and ethnicity, and Poisson regression including catchment-area level risk factors. For the prevention of psychosis two main lessons can be learned: services focused on early intervention should not have an upper age limit as half of all female (and 32% of male) cases present after age 35, and future examinations of variance should focus on socioenvironmental and not geographical determinants.

